# Effects of reproductive experience on cost-benefit decision making in female rats

**DOI:** 10.3389/fnbeh.2024.1304408

**Published:** 2024-01-29

**Authors:** Mojdeh Faraji, Omar A. Viera-Resto, Barry Setlow, Jennifer L. Bizon

**Affiliations:** ^1^Department of Psychiatry, University of Florida, Gainesville, FL, United States; ^2^Center for Addiction Research and Education, University of Florida, Gainesville, FL, United States; ^3^McKnight Brain Institute, University of Florida, Gainesville, FL, United States; ^4^Department of Neuroscience, University of Florida, Gainesville, FL, United States

**Keywords:** pregnancy, mating, reproduction, risk taking, impulsivity, decision making

## Abstract

Many individuals undergo mating and/or other aspects of reproductive experience at some point in their lives, and pregnancy and childbirth in particular are associated with alterations in the prevalence of several psychiatric disorders. Research in rodents shows that maternal experience affects spatial learning and other aspects of hippocampal function. In contrast, there has been little work in animal models concerning how reproductive experience affects cost–benefit decision making, despite the relevance of this aspect of cognition for psychiatric disorders. To begin to address this issue, reproductively experienced (RE) and reproductively naïve (RN) female Long-Evans rats were tested across multiple tasks that assess different forms of cost–benefit decision making. In a risky decision-making task, in which rats chose between a small, safe food reward and a large food reward accompanied by variable probabilities of punishment, RE females chose the large risky reward significantly more frequently than RN females (greater risk taking). In an intertemporal choice task, in which rats chose between a small, immediate food reward and a large food reward delivered after a variable delay period, RE females chose the large reward less frequently than RN females. Together, these results show distinct effects of reproductive experience on different forms of cost–benefit decision making in female rats, and highlight reproductive status as a variable that could influence aspects of cognition relevant for psychiatric disorders.

## 1 Introduction

Reproductive experience (i.e., mating, pregnancy, childbirth, and/or parenting) is associated with a range of somatic, hormonal, and neural adaptations that prepare organisms to create and raise their offspring (Mastorakos and Ilias, 2000; Tal and Taylor, 2000; Tan and Tan, 2013; Hoyt and Falconi, 2015; Johnson and Cipolla, 2015; Mizuno et al., 2017; Bhatia and Chhabra, 2018; Reyes et al., 2018; Wen et al., 2019). The female mammalian brain in particular undergoes remarkable alterations during the peripartum period. Studies in rodents show shifts in corticotropin-releasing hormone (CRH), opioid, oxytocin, and dopamine signaling, as well as changes in gene expression, along with altered progesterone and estrogen profiles during the peripartum period (Brunton and Russell, 2008; Kinsley and Amory-Meyer, 2011; Robinson et al., 2011; Alcántara-Alonso et al., 2017; Shnitko et al., 2017; Brunton, 2019; Cárdenas et al., 2020). Circulating hormone levels in particular are dramatically altered during pregnancy due to changes in ovarian activity. As estradiol and progesterone can easily pass through the blood–brain-barrier, CNS levels of gonadal hormones are also altered during pregnancy (Bernal and Paolieri, 2022). In humans, there are changes in neural organization and resting state brain activity (Hoekzema et al., 2022), as well as alterations in gray matter volume and cortical thickness during pregnancy (Rehbein et al., 2022) and early postpartum (Chechko et al., 2022; Servin-Barthet et al., 2023). Many of these brain changes (particularly those observed in rodents) have been linked to offspring-relevant behaviors, including gestation, birth, lactation, pair bonding/affiliation, and maternal aggression (Leuner et al., 2010; Hahn-Holbrook et al., 2011; Arévalo and Campbell, 2020; Walter et al., 2021). The peripartum period is also associated with changes in vulnerability to psychiatric disorders, however, including depression (Skalkidou et al., 2012; Pawluski et al., 2017; Barba-Müller et al., 2019; Morgan et al., 2021; Osborne et al., 2022; Worthen and Beurel, 2022), anxiety, puerperal psychosis, obsessive-compulsive disorder, and post-traumatic stress disorder (Miller et al., 2015a,b; Meltzer-Brody et al., 2018; Barba-Müller et al., 2019). The causes of such vulnerability changes are unclear and likely multifaceted (e.g., genetic or environmental predispositions, hormonal fluctuations, peripartum shifts in socioeconomic conditions). As such, the use of animal models can provide experimental control that can help to identify biological and/or environmental contributions to the effects of reproductive experience on behavior.

Despite numerous studies documenting links between reproductive experience and changes in risks of psychiatric disorders, most preclinical research on the consequences of reproductive experience (outside that tied explicitly to offspring-directed behaviors) has focused on the hippocampus and hippocampal functions such as learning and memory (Pawluski et al., 2006; Maeng and Shors, 2012; Roes and Galea, 2016; Shors, 2016; Eid et al., 2019; Moses-Kolko et al., 2021). In contrast, other brain systems implicated in psychiatric disorders (e.g., prefrontal cortex and amygdala) and associated aspects of cognition (e.g., executive functions, including decision making) have received less attention (Leuner et al., 2014; Albin-Brooks et al., 2017) despite the importance of executive functions in maternal caregiving (Harris et al., 2021; Nordenswan et al., 2021). In humans, both increased (Kim et al., 2018) and decreased (Hoekzema et al., 2017) gray matter volume and thickness of prefrontal cortex have been reported following pregnancy and childbirth. In addition, the default mode network (DMN), a closely interconnected system of brain regions (including prefrontal cortex) representing the brain’s baseline activity that is involved in self-perception and social cognition, is functionally altered during pregnancy and after childbirth. Hypoactivity in the DMN during working memory (Bak et al., 2020) and altered coherence of DMN across pregnancy in response to infant cues are examples of such changes (Hoekzema et al., 2022). Alterations in decision making are prevalent in a range of psychiatric disorders, including those linked to reproductive experience (Cáceda et al., 2014; Teng et al., 2016; Morris, 2018; Mukherjee et al., 2020; Valyan et al., 2020; Purcell et al., 2022). As such, a better understanding of the effects of reproductive experience on executive functions/decision making could lead to new insights into the etiology of peripartum changes in risks of psychiatric disorders.

The majority of research on the effects of reproductive experience on brain and behavior has focused on the peripartum period (i.e., during pregnancy and/or early child rearing). A growing body of evidence, however, suggests that reproductive experience can have effects that far outlast this period (Hoekzema et al., 2017; Duarte-Guterman et al., 2019). For example, a neuroimaging study in humans revealed reductions in gray matter in a cohort of primiparous mothers that persisted for 6 years postpartum (Martínez-García et al., 2021), and in another study, gray matter volume in mid-life was positively associated with number of children (Schelbaum et al., 2021). In addition, although risks for psychiatric disorders may be most prevalent in the near peripartum period, risks can remain elevated for up to 3 years following childbirth (Putnick et al., 2020), indicating the potential for long-lasting effects of reproductive experience. It can be challenging to separate intrinsic biological factors from social/environmental factors concerning the post-partum duration of brain and behavior changes in humans, due to the extended period of child rearing and attendant socioeconomic and cultural stressors. Studies in animals, however, in which lifespans are shorter and environmental conditions can be better controlled, can help to address these factors.

The goal of the current studies was to begin to address these gaps in our understanding of the effects of reproductive experience on cognition. To do so, we evaluated the long-term (up to 6 months) effects of reproductive experience in female rats on several forms of cost–benefit decision making that have been linked previously to differences in risks of psychiatric disorders (Linnet et al., 2011; Gowin et al., 2013; Kaye et al., 2013; Steinglass et al., 2017; Amlung et al., 2019; Mitchell, 2019; Soutschek et al., 2022). On each task, reproductively-experienced and-naïve rats were given choices between a small reward associated with no cost and a large reward associated with various possible costs (delay to delivery, explicit punishment, reward omission). Complementary experiments were conducted to evaluate effects of reproductive experience on food motivation, shock reactivity threshold, and cognitive flexibility.

## 2 Methods

### 2.1 Subjects

Female (*n* = 32) Long-Evans rats, 50 days of age upon arrival from Charles River Laboratories, were individually housed on a 12 h light/dark cycle (lights on at 0700), maintained at a consistent temperature of 25°, and had access to water and 2919 Teklad irradiated global 19% protein chow. Rats were allowed to acclimate to vivarium conditions for at least 1 week before the start of any procedures.

Experiments were conducted in two cohorts. In each cohort (females, *n* = 16), half of the females (*n* = 8) were paired with a male Long-Evans rat of the same age while the rest of the females (*n* = 8) remained unpaired. Males were removed from the cages once pregnancies were confirmed. For the rest of this report, the paired (mated) rats are referred to as reproductively experienced (RE), and the unpaired rats as reproductively naïve (RN). RE females gave birth, nursed, and pups were weaned after 21 days. One week after weaning, RE females were paired again with a different male from the same group (to minimize potential effects of specific male mates on females’ reproductive experience). Males were removed again once pregnancies were confirmed, and were not used further in the experiments. Females gave birth, nursed, and pups were weaned after 21 days. One female from the first cohort did not become pregnant in either round of pairing and was excluded from the study. One female from the second cohort was eliminated from the study due to infanticide in both of her litters.

All rats had access to food and water *ad libitum* until 1 week after the last weaning, when they were put on food restriction. Water access remained *ad libitum* at all times. Behavioral experiments started once rats reached 85% of their free-feeding weights, and rats were individually housed throughout the duration of the behavioral experiments. The behavioral tasks in which the rats were tested proceeded in the following order for both cohorts: intertemporal choice, progressive ratio schedule of reinforcement, risky decision making, probabilistic reversal learning, reward omission vs. punishment, and shock reactivity threshold, and took approximately 6 months to complete. All procedures were conducted in accordance with the University of Florida Institutional Animal Care and Use Committee and adhered to the guidelines of the National Institutes of Health.

### 2.2 Apparatus

Behavioral testing was conducted in eight standard operant chambers (Coulbourn Instruments). Chambers were contained in sound-attenuating cubicles, and were computer controlled through Graphic State 4.0 software (Coulbourn Instruments). Locomotor activity was monitored via infrared motion detectors installed on the ceilings of the chambers. Each operant chamber was equipped with a food trough containing a photobeam sensor to detect nosepokes into the trough, two retractable levers (one on each side of the food trough), a feeder installed on the outside wall of the chamber and connected to the food trough to deliver 45 mg purified ingredient rodent food pellets (Test Diet 5TUL), and a stainless steel floor grate connected to a shock generator that could deliver scrambled footshocks. Each sound-attenuating cubicle included a house light mounted on the rear wall (outside of the operant chamber).

### 2.3 Behavioral procedures

#### 2.3.1 Risky decision-making task

Prior to the start of all operant behavioral testing, rats were trained on a sequence of shaping protocols to learn how to retrieve food from the food trough, nosepoke in the food trough to initiate a trial, and press the levers to obtain food. Shaping procedures are described in detail in Blaes et al. (2022).

**Figure 1:**
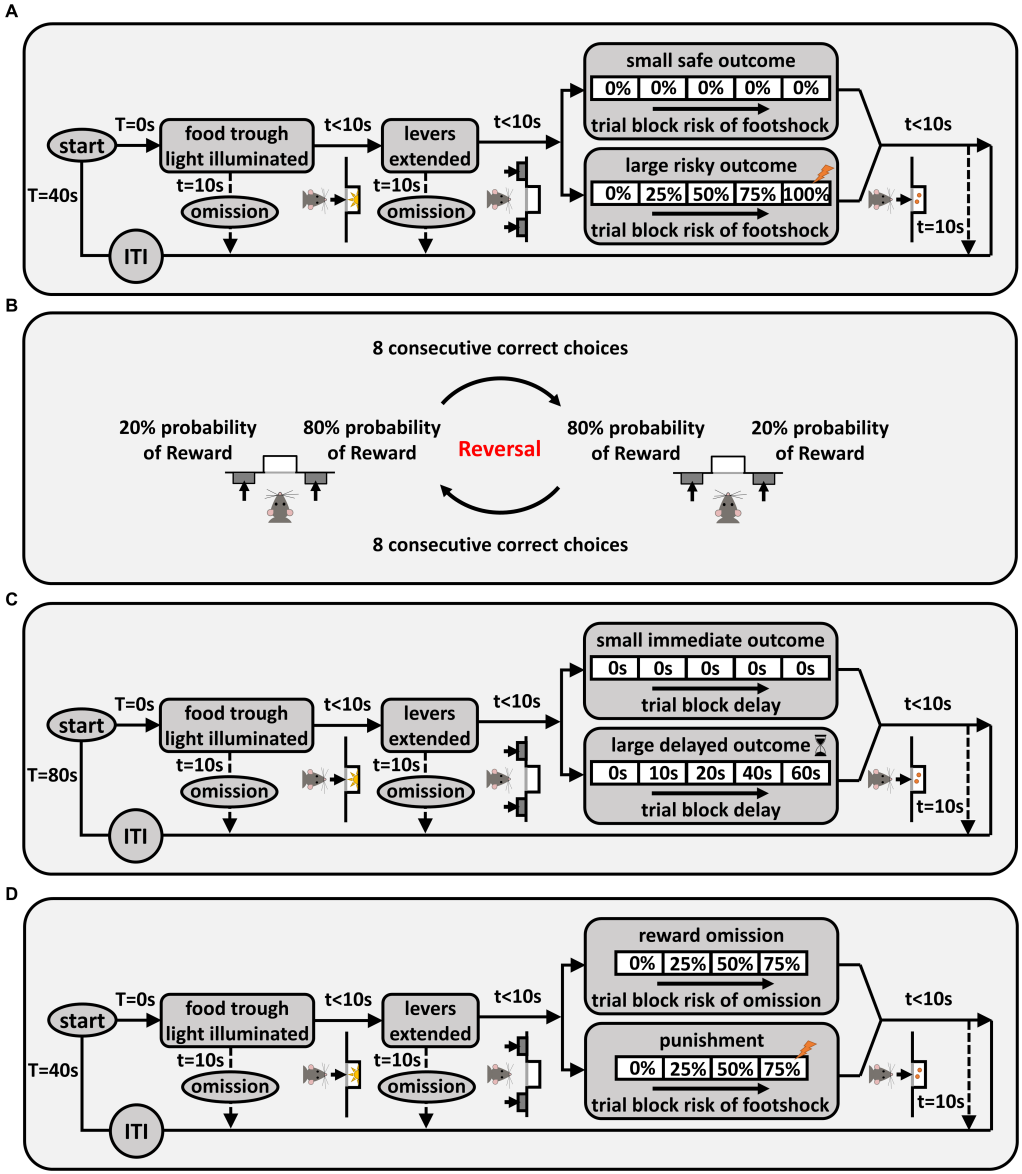
Behavioral task schematics. **(A)** Risky decision-making task (RDT). Each trial was initiated by nosepoking into the food trough, and pressing a lever led to delivery of food pellet(s). If the large reward lever was chosen, delivery of this reward was accompanied by probabilistic shock delivery. There were five blocks of trials, and the probability of shock increased as blocks proceeded. “T” denotes the total time spent in the trial and “t” denotes the time spent in each state of the trial. **(B)** Probabilistic reversal learning. The left/right positions of the advantageous (80% chance of reward) and disadvantageous (20% chance of reward) levers were reversed once rats chose the advantageous side 8 times consecutively. **(C)** Intertemporal choice task. Each trial was initiated by nosepoking into the food trough, and pressing a lever led to delivery of food pellet(s). If the large reward lever was chosen, food delivery was preceded by a delay. There were five blocks of trials in the task, in which the delay to large reward delivery increased across successive blocks. **(D)** Reward omission vs. punishment task (ROVP) Rats had to decide between risk of reward omission vs. risk of punishment. Each trial was initiated by nosepoking into the food trough, and pressing the levers led to either delivery of reward alone, reward omission, or a reward accompanied by a footshock. There were four blocks of trials in the ROVP task, in which the probability of contingencies (reward omission or shock accompanying the reward) increased across successive blocks.

In the risky decision-making task (RDT) (Simon et al., 2009), rats made discrete-trial choices between two levers. Each trial started with illumination of the house light and the food trough light. A nosepoke into the food trough during this time caused the food trough light to be extinguished and either one lever (on forced-choice trials) or both levers (on free-choice trials) to extend into the chamber. A failure to nosepoke within 10 s was counted as an omission. A press on one lever (the “small, safe” lever) yielded a single food pellet, while a press on the other lever (the “large, risky” lever) yielded two food pellets and was accompanied by varying probabilities of a mild footshock (1.0 s in duration). A failure to press either lever within 10 s resulted in termination of the trial and marked it as an omission. Lever presses were followed by retraction of the lever(s), illumination of the food trough light, and delivery of food pellets. The food trough light was extinguished upon retrieval of the pellets or after 10 s, whichever came first. Trials were separated by an intertrial interval (ITI) in which the house light was extinguished. Each session was comprised of 5 blocks of trials, with different probabilities of shock accompanying the large reward in each block (0, 25, 50, 75, 100%). Figure 1A depicts a schematic of the RDT. Each block of trials began with 8 forced-choice trials (4 for each lever) in which only one lever was extended. The purpose of these trials was to remind rats of the probability of shock in that block. The forced-choice trials were followed by 10 free-choice trials in which both levers were extended. Sessions in the RDT were 60 min in duration and consisted of 90 trials, each 40 s long. The left/right positions of the small and large reward levers were randomized across rats, but remained consistent for each rat over the course of the task. Shock intensities were 150 μA for the first cohort and 250 μA for the second cohort, but were the same for all rats within each cohort. Rats were trained on the RDT until stable choice performance emerged (see section 2.4 for definition of stable performance).

#### 2.3.2 Progressive ratio schedule of reinforcement

Motivation to pursue food was tested in a progressive ratio schedule of reinforcement task in the same operant chambers used for the RDT. In this task, a single lever (the same lever used as the “small, safe” lever in the RDT) was extended into the chamber and remained extended for the duration of the session. Presses on the lever earned a single food pellet reward; however, the number of presses required to earn subsequent food rewards increased with each successive reward earned according to the following sequence: 1, 3, 6, 10, 15, 20, 25, 32, 40, 50, 62, 77, 95, 118, 145, 175, 205, 235, 265, 295, 325, 355. In any trial, failure to earn a food pellet within 10 min resulted in termination of the session. Testing in this task continued for 10 sessions (1 session/day).

#### 2.3.3 Shock reactivity threshold

To determine whether reproductive experience affects reactivity to shock, rats were tested in a shock reactivity threshold assay (Bonnet and Peterson, 1975; Orsini et al., 2017a). Rats were placed in a standard operant chamber (a different chamber from that in which they underwent the other behavioral tests) and were initially administered a 400 μA shock to attenuate spontaneous movement in the chamber. Subsequent shocks (1.0 s in duration) were delivered at 10 s intervals, beginning at 50 μA. Responses to the shocks were recorded by a trained observer. Shock reactivity criteria were defined as either (1) flinch of a paw or a startle response, (2) elevation of one or two paws, or (3) rapid movement of three or all paws. If a shock did not elicit any of the three behaviors, the intensity was increased by 25 μA. If a response was elicited, the intensity was reduced by 25 μA. This procedure continued until the rat was responsive to an intensity 3 times and not responsive to the intensity 25 μA lower twice in a row (following a “yes-no-yes-no-yes” pattern). If a rat showed three consecutive reactions to 50 μA, then 50 μA was recorded as the reactivity threshold for that rat. Each rat was tested twice (on two consecutive days) and the results were averaged.

#### 2.3.4 Probabilistic reversal learning

To evaluate effects of reproductive experience on behavioral flexibility, rats were tested on a probabilistic reversal learning task (PRT). In this task (which was modeled on Dalton et al., 2014, and conducted in the same operant chambers used for the RDT), rats were presented with two levers that differed in the probability with which a press yielded a food reward (Figure 1B). Each 15 s trial began with illumination of the house light for 3 s, followed by extension of both levers. Pressing either lever led to retraction of both levers, illumination of the food trough light, and delivery of a single food pellet (on rewarded trials), followed by an ITI. On trials that were not rewarded (no food delivery), a lever press was immediately followed by the ITI. Failure to press either lever within 10 s of their extension caused the levers to be retracted and the trial counted as an omission. At the start of each session, the probability of food delivery on one of the levers (left or right, randomized across sessions) was 80% (advantageous side) and the probability on the other was 20% (disadvantageous side). Eight consecutive choices of the advantageous side (excluding trial omissions) caused the probabilities of reward delivery on the two levers to reverse, such that the previously advantageous side became disadvantageous and vice versa, and rats had to learn the new reward contingencies. This criterion continued throughout the 200 trials in a session, such that a rat could achieve multiple reversals within a session. Each session was 50 min in duration, and rats were tested in the task for 10 consecutive sessions (1 session/day).

#### 2.3.5 Intertemporal choice task

Preference for immediate vs. delayed gratification was assessed in an intertemporal choice task (Evenden and Ryan, 1996; Hernandez et al., 2020). In this task (conducted in the same operant chambers used for the RDT), rats made discrete-trial choices between a small, immediate reward and a large, delayed reward. Each trial started with illumination of the house light and the food trough light. A nosepoke into the food trough during this time caused the food trough light to be extinguished and either one lever (on forced-choice trials) or both levers (on free-choice trials) to extend into the chamber. A failure to nosepoke within 10 s was counted as an omission. Pressing the small reward lever led to illumination of the food trough light and immediate delivery of a food pellet. Pressing the large reward lever led to the delay phase in which, after the delay timer expired, the food trough light was illuminated and three food pellets were delivered. A failure to press a lever within 10 s was counted as an omission. Lever presses were followed by retraction of the lever(s). The food trough light was extinguished upon food retrieval or after 10 s, whichever came first. Trials were separated by an ITI in which the house light was extinguished. Each session was comprised of 5 blocks of trials with different delay durations (0, 10, 20, 40, 60 s). Figure 1C shows a schematic of the task. Each block of trials began with 2 forced-choice trials (one for each lever) in which only one lever was extended. The purpose of these trials was to remind rats of the duration of the delay in that block. The forced-choice trials were followed by 10 free-choice trials in which both levers were extended. Sessions in the intertemporal choice task were 80 min in duration and consisted of 60 trials, each 80 s long. The left/right positions of the small and large reward levers were randomized across rats, but remained consistent for each rat over the course of the task. Rats were trained on the intertemporal choice task until stable choice performance emerged (see section 2.4 for definition of stable performance).

#### 2.3.6 Reward omission vs. punishment task

To evaluate preference for different types of risks, rats were tested on a novel task in which they made choices between two parallel contingencies: risk of reward omission vs. risk of punishment (the “Reward Omission Vs. Punishment” (ROVP) task). Each trial in this task started with illumination of the house light and the food trough light. A nosepoke into the food trough during this time caused the food trough light to be extinguished and either one lever (on forced-choice trials) or both levers (on free-choice trials) to extend into the chamber. A failure to nosepoke within 10 s was counted as an omission. A press on one lever (the “omission” lever) yielded a single food pellet that was delivered with varying probabilities, while a press on the other lever (the “punishment” lever) always yielded a single food pellet but was accompanied by varying probabilities of a mild footshock. A failure to press either lever within 10 s resulted in termination of the trial and was counted as an omission. Lever presses were followed by retraction of the lever(s), illumination of the food trough light, and delivery of a food pellet. The food trough light was extinguished upon retrieval of the pellet or after 10 s, whichever came first. Trials were separated by an ITI in which the house light was extinguished. Each session was comprised of 4 blocks of trials with different probabilities of contingencies (0, 25, 50, and 75% probability of reward omission or shock delivery). Figure 1D shows a schematic of the ROVP task. Each block of trials began with 8 forced-choice trials (4 for each lever) in which only one lever was extended. The purpose of these trials was to remind rats of the probability of contingencies in that block. The forced-choice trials were followed by 10 free-choice trials in which both levers were extended. Sessions in the ROVP task were 48 min long and consisted of 72 trials, each 40 s in duration. The left/right positions of the “omission” and “punishment” levers were randomized across rats, but remained consistent for each rat over the course of the task. Shock intensities were the same for all rats within each of the two cohorts (200 and 350 μA for the first and second cohort, respectively, 1.0 s in duration). Rats were trained on the ROVP task until stable choice performance emerged (see section 2.4 for definition of stable performance).

### 2.4 Data analysis

Data were collected and processed using custom protocols and analysis templates in Graphic State 4.0. Statistical analyses were conducted and graphs created using GraphPad Prism 9. To evaluate stable performance in the decision-making tasks, two-factor repeated-measures analyses of variance (ANOVA) with session and trial block as within-subjects factors were conducted on choice data across three (RDT and ROVP) or five (intertemporal choice task) consecutive sessions within each of the two groups separately (RE and RN). Stability was defined as the absence of a significant main effect of session or session × trial block interaction.

The primary measures of interest in the decision-making tasks were as follows: in the RDT, percentage of large, risky reward lever presses in each block (out of the total number of trials completed); In the intertemporal choice task, percentage of large, delayed reward lever presses in each block (out of the total number of trials completed); In the ROVP task, percentage of either reward omission lever presses, punishment lever presses, or no lever presses (omitted trials), as a proportion of the total possible number of trials in each block (10/block). These primary measures were evaluated using two-factor repeated-measures ANOVAs, with reproductive experience as a between-subjects factor and trial block as a within-subjects factor, on data averaged across stable sessions. In the intertemporal choice task, choice indifference points were also calculated, using a hyperbolic decay function fitted to each rat’s percent choice of the large reward at each delay block. The resulting formula was then used to estimate the delay at which choice of the large reward was equal to 50% (the delay at which the rat was equally likely to choose the large or small reward).

To examine the immediate effects of aversive outcomes on trial-by-trial choice behavior under conditions in which significant differences in choice behavior were observed, win-stay/lose-shift analyses were conducted on data from the RDT and ROVP tasks as in Orsini et al. (2017a). Trials on which choices were not accompanied by a contingency (footshock on the large reward lever in the RDT and punishment lever in the ROVP, and reward omission on the omission lever in the ROVP) were considered a “win,” and trials on which choices were accompanied by a contingency (footshock or reward omission) were considered a “loss.” Win-stay was defined as when a win trial was followed by choice of the same option on the next trial. Lose-shift was defined as when a lose trial was followed by a switch to the opposite choice (to the safe choice in the RDT and to the other lever in the ROVP) on the next trial. Win-stay performance was measured by dividing the number of win-stay trials by the total number of win trials, and lose-shift performance was measured by dividing the number of lose-shift trials by the total number of lose trials. Both measures were calculated from free-choice trials only. Results were compared between the RE and RN female groups using a two-factor repeated-measures ANOVA, with reproductive experience as a between-subjects factor and trial block as a within-subjects factor, on data averaged across stable sessions.

Progressive ratio and probabilistic reversal learning data were evaluated using a two-factor repeated-measures ANOVA, with reproductive experience as a between-subjects factor and session as a within-subjects factor, across the 10 days of task performance. Shock reactivity threshold results were analyzed using a Welch’s *t*-test, with reproductive experience as the grouping factor.

Ancillary measures in the decision-making tasks, such as locomotor activity, shock reactivity (locomotor activity during the shock delivery period), and omissions during free choice trials, were analyzed using Welch’s *t*-tests, comparing data averaged across trial blocks during the stable sessions of performance. Latencies to press levers during forced- and free-choice trials were used to assess motivation to pursue the outcomes associated with each choice. These data were analyzed using a three-factor repeated-measure ANOVA, with reproductive experience as a between-subjects factor and lever identity and trial block as within-subjects factors, on data averaged across stable sessions. The Greenhouse–Geisser correction was used to account for violations of sphericity in ANOVAs, and for all analyses, *p*-values less than or equal to 0.05 were considered significant.

## 3 Results

### 3.1 Risky decision-making

**Figure 2:**
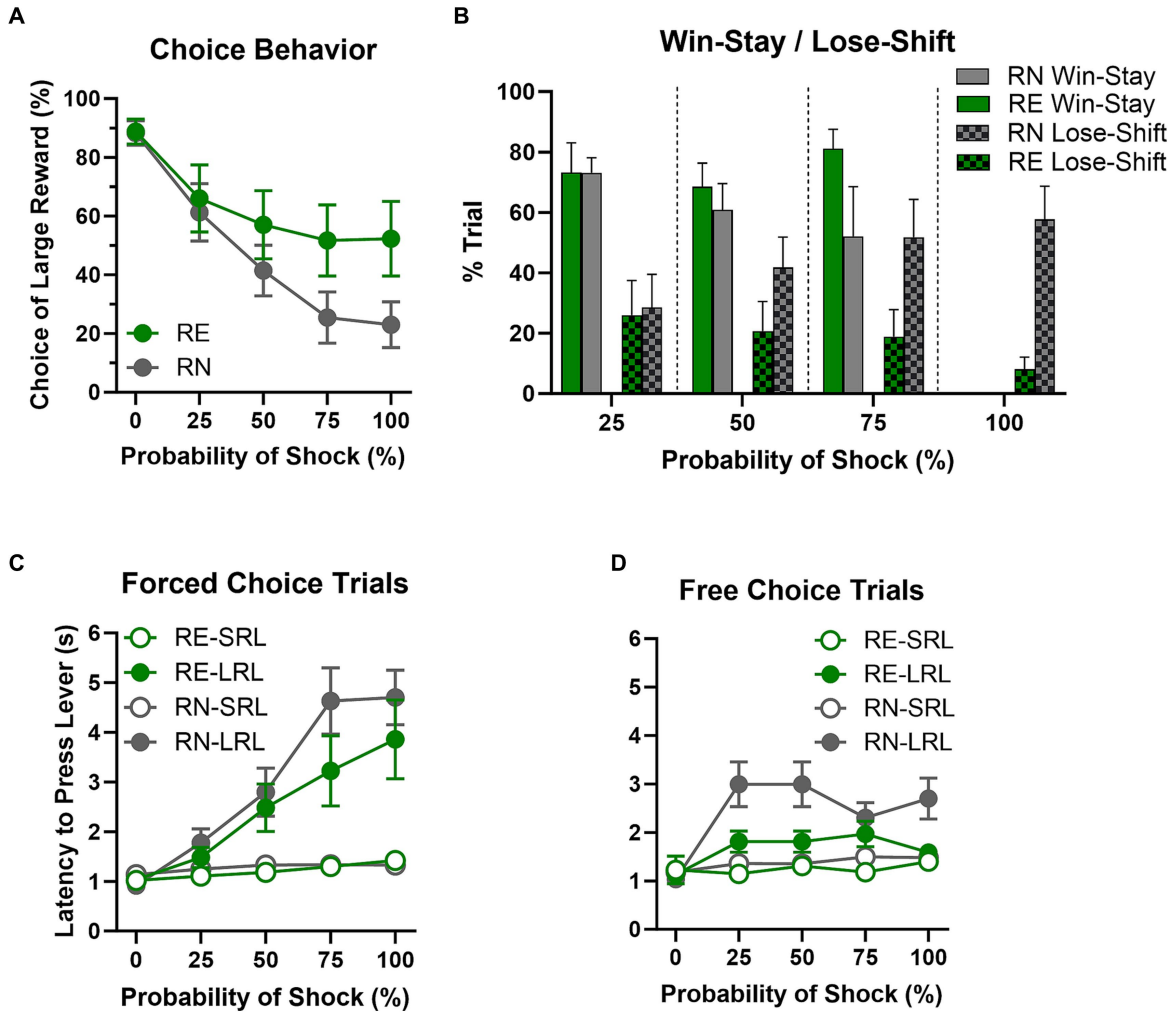
Risky decision making task performance. **(A)** Choice behavior. RE females chose the large risky reward more frequently than RN females at higher shock probabilities. **(B)** Win-stay/lose-shift. There were no main effects or interaction on win-stay performance. Lose-shift performance, however, was less frequent in RE compared to RN females as shock probabilities increased. **(C,D)** Latencies to press levers. On forced-choice trials, RE and RN females’ latencies to press the small reward lever (SRL) were comparable, and differences between the group latencies in pressing the large reward lever (LRL) did not reach statistical significance. On free-choice trials, however, RE females showed shorter latencies than RN females at higher shock probabilities. Data are represented as means ± SEM.

Rats were tested on the RDT until stable performance emerged (first cohort, 36 sessions; second cohort, 40 sessions). Analysis of % choice of the large, risky reward using a two-factor repeated measures ANOVA (Group × Shock Probability) revealed a main effect of Shock Probability [*F*(2.32, 63.26) = 36.71, *p* < 0.01], such that preference for the large reward declined as probability of shock increased, but no main effect of Group [*F*(1, 28) = 1.91, *p* = 0.18]. There was, however, a significant interaction between Shock Probability and Group [*F*(4, 109) = 3.64, *p* < 0.01], such that RE females chose the large reward more frequently than RN females at higher shock probabilities (Figure 2A).

Analysis of win-stay results using a two-factor repeated measures ANOVA (Group × Shock Probability; Figure 2B) showed that the likelihood of making the same choice after a “win” was unaffected by Group [*F*(1, 22) = 1.52, *p* = 0.23], Shock Probability [*F*(1.63, 27.64) = 1.15, *p* = 0.32], or the interaction of the two variables [Group × Shock Probability, *F*(2, 34) = 1.34, *p* = 0.28]. In contrast, the same analysis conducted on lose-shift results showed that the likelihood of switching to the opposite choice after a loss (shock) was unaffected by Shock Probability [*F*(2.84, 50.11) = 2, *p* = 0.13], but there was a main effect of Group [*F*(1, 23) = 4.41, *p* = 0.05] and a significant interaction between the two variables [*F*(3, 53) = 3.37, *p* = 0.03], such that as shock probability increased across blocks, RN females were more and RE females less likely to switch their choice to the opposite lever following a loss (shock).

Females’ latencies to press levers on both forced-choice and free-choice trials were analyzed using a three-factor repeated-measure ANOVA (Group × Lever × Shock Probability). On forced-choice trials (Figure 2C), latencies were longer at higher shock probabilities [*F*(2.10, 113.5) = 46.24, *p* < 0.01] and on the large compared to the small reward lever [*F*(1, 57) = 36.58, *p* < 0.01], particularly at higher shock probabilities [Shock Probability × Lever: *F*(4, 217) = 34.18, *p* < 0.01]. Response latencies did not differ between RN and RE groups, however [Group: *F*(1, 57) = 1.12, *p* = 0.3; Group × Shock Probability: *F*(4, 217) = 0.86, *p* = 0.49; Group × Lever: *F*(1, 57) = 0.61, *p* = 0.44; Group × Shock Probability × Lever: *F*(4, 217) = 1.59, *p* = 0.18]. On free choice trials (Figure 2D), rats had longer latencies at higher shock probabilities [*F*(1.83, 75.43) = 20.46, *p* < 0.01] and when choosing the large reward [Lever: *F*(1, 56) = 25.71, *p* < 0.01], particularly at higher shock probabilities [Shock Probability × Lever: *F*(4, 165) = 12.52, *p* < 0.01]. There were no Group × Lever [*F*(1, 56) = 2.72, *p* = 0.14] or Group × Lever × Shock Probability [*F*(4, 165) = 1.87, *p* = 0.12] interactions; however, there was a main effect of Group [*F*(1, 56) = 4.25, *p* = 0.04] and a Group × Shock Probability interaction [*F*(4, 165) = 2.79, *p* = 0.03], such that RE females had shorter latencies than RN females, particularly at higher shock probabilities.

A Welch’s *t*-test was used to evaluate locomotor activity during the ITI, which revealed no difference between the RE and RN females [*t*(26.49) < 0.01, *p* = 0.99]. The same analysis conducted on shock reactivity during the task (locomotor activity during the shock delivery period) and number of free-choice trial omissions also revealed no group differences [shock reactivity: *t*(18) = 0.32, *p* = 0.75; trial omissions: *t*(27.93) = 1.25, *p* = 0.22]. Table 1 shows mean (SEM) values across trial blocks between female groups.

**Table 1:** Mean (± standard error of the mean) locomotor activity and omissions.

	Locomotor activity (locomotor units/ITI)	Shock reactivity (locomotor units/shock)	Trial omissions
RDT			
RE females	13.26 (17.03)	1.80 (1.23)	4.48 (6.22)
RN females	13.21 (15.39)	2.00 (1.51)	7.50 (6.92)

	Locomotor activity (locomotor units/ITI)	Trial omissions
Intertemporal choice task		
RE females	37.50 (26.82)	4.49 (8.34)
RN females	39.48 (41.75)	13.63 (19.05)

### 3.2 Progressive ratio schedule of reinforcement

**Figure 3:**
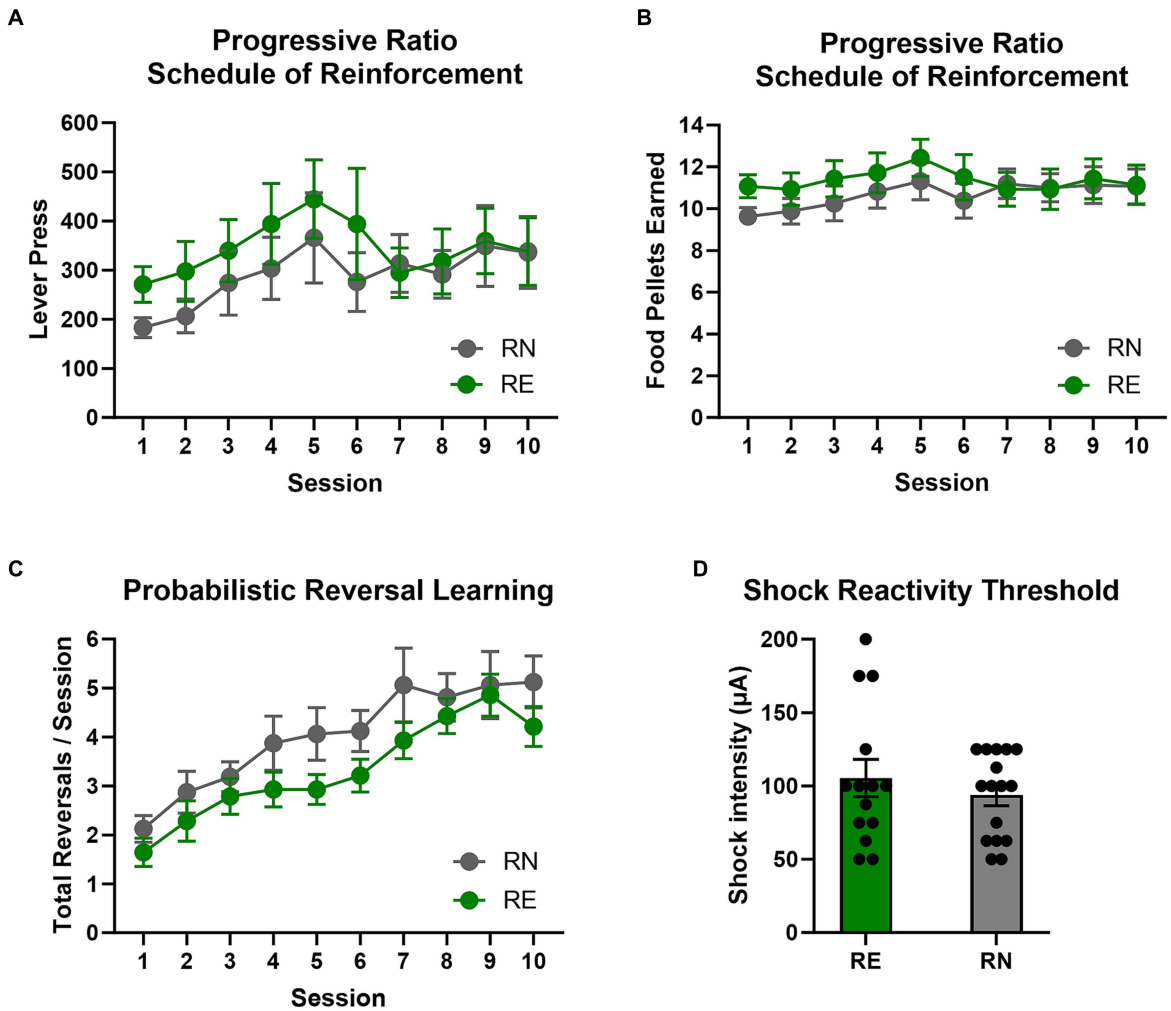
Progressive ratio, reversal learning, and shock reactivity. **(A,B)** Progressive ratio schedule of reinforcement. There were no group differences in the number of lever presses or food pellets earned between RE and RN females, suggesting equivalent motivation to obtain food. **(C)** Probabilistic reversal learning. There were no group differences in the number of reversals completed per session in the probabilistic reversal learning task. **(D)** Shock reactivity threshold. Groups did not differ in shock reactivity threshold. Data are represented as means ± SEM.

To determine whether the effects of reproductive experience on RDT performance could be due to broader differences in willingness to incur costs to obtain food, rats were tested on a progressive ratio schedule of reinforcement across 10 consecutive sessions (Figures 3A,B). A two-factor repeated measures ANOVA (Session × Group) comparing the number of lever presses per session revealed a main effect of Session [*F*(1.63, 45.72) = 3.46, *p* = 0.05], but no main effect of Group [*F*(1, 28) = 0.48, *p* = 0.49] or interaction between the two variables [*F*(9, 252) = 0.71, *p* = 0.70]. A comparable analysis conducted on the number of food pellets earned per session revealed similar results [Session: *F*(1.43, 39.96) = 2.40, *p* = 0.12; Group: *F*(1, 28) = 0.45, *p* = 0.51; Session × Group: *F*(9, 252) = 1.17, *p* = 0.32]. These data suggest that an increase in food motivation does not account for greater preference for the large, risky reward in RE females in the RDT.

### 3.3 Probabilistic reversal learning

To determine whether effects of reproductive experience on RDT performance were associated with differences in behavioral flexibility, rats were tested on the probabilistic reversal learning task (Figure 3C). A two-factor repeated-measures ANOVA (Group × Session) conducted on the number of completed reversals per session revealed a main effect of Session [*F*(2.56, 71.63) = 18.21, *p* < 0.01] such that rats completed more reversals as sessions progressed, but there was neither a main effect of Group [*F*(1, 28) = 2.28, *p* = 0.14] nor a Session × Group interaction [*F*(9, 252) = 0.50, *p* = 0.87]. A similar analysis was conducted on the number of trials completed per reversal (representing the rapidity with which reversals were learned), showing that rats met the reversal criterion in fewer trials as sessions progressed [Session: *F*(4.03, 112.7) = 12.87, *p* < 0.01], but revealing comparable results between groups [Group: *F*(1, 28) = 1.82, *p* = 0.19; Session × Group: *F*(9, 252) = 0.94, *p* = 0.49]. Analysis of the number of total omitted trials per session revealed neither main effects of Group [*F*(1, 28) = 0.60, *p* = 0.44] or Session [*F*(1.94, 54.28) = 2.57, *p* = 0.09], nor a Session × Group interaction [*F*(9, 252) = 1.10, *p* = 0.37]. Considered together, these data show that reproductive experience in females does not affect reversal learning, and suggest that reduced behavioral flexibility does not account for greater choice of the large, risky reward in RE rats across blocks in the RDT.

### 3.4 Shock reactivity threshold

Rats were tested for their shock reactivity threshold to determine whether the effects of reproductive experience on RDT performance could be due to differences in shock sensitivity (Figure 3D). Comparison of reactivity thresholds using a Welch’s *t*-test revealed no difference between RE and RN groups [*t*(20.88) = 0.80, *p* = 0.44].

### 3.5 Intertemporal choice task

**Figure 4:**
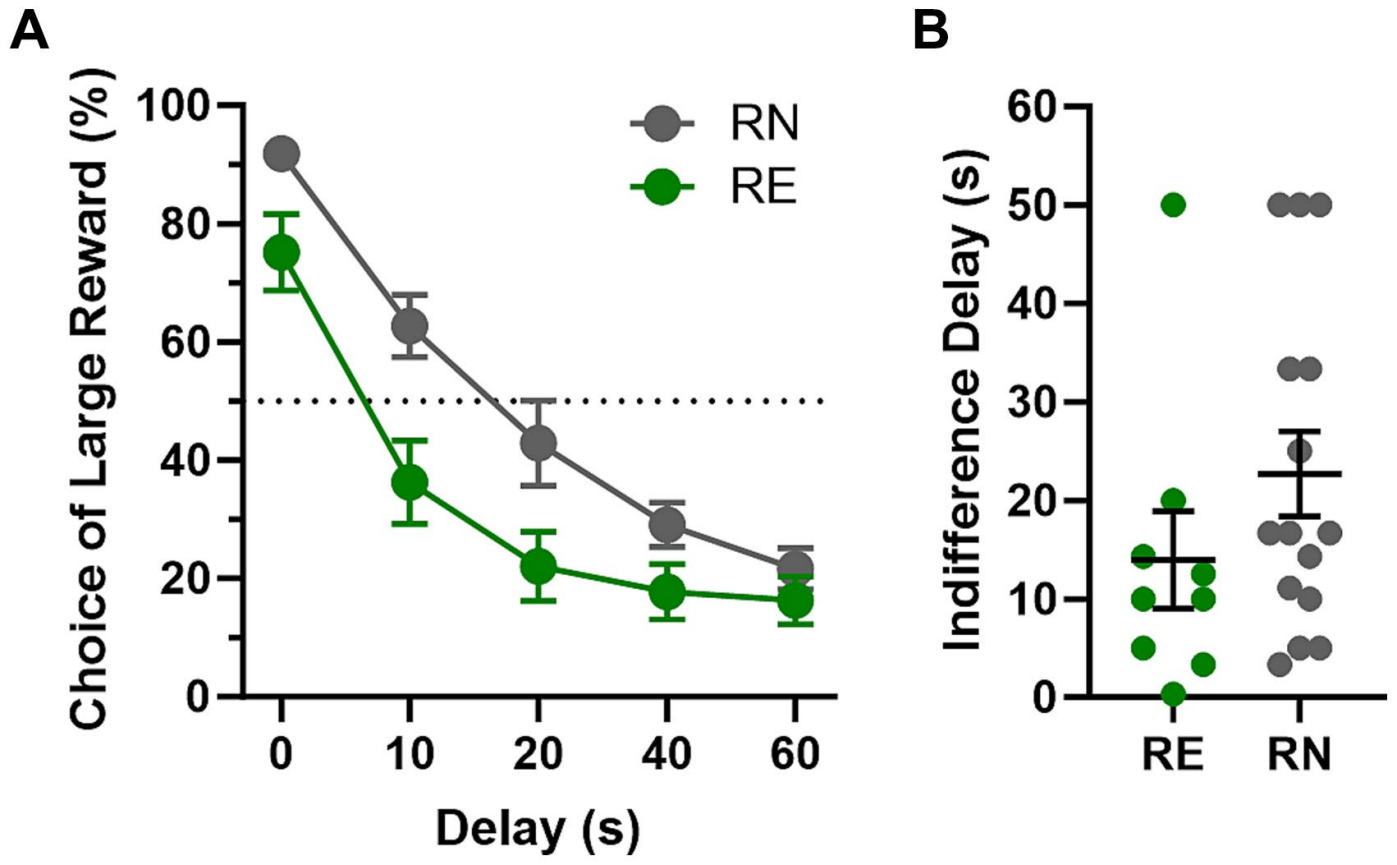
Intertemporal choice task performance. **(A)** Choice behavior. RE females showed reduced preference for the large, delayed reward compared to RN females, particularly at short delays. **(B)** Indifference delay. RE females showed shorter indifference delays (the theoretical delay to large reward delivery at which preference between the large, delayed and small, immediate reward is equivalent) compared to RN females. Data are represented as means ± SEM.

Rats were tested on the intertemporal choice task until stable choice behavior emerged (first cohort, 31 sessions; second cohort, 21 sessions). Comparison of choice of the large, delayed reward in RN and RE rats during stable performance using a two-factor repeated-measures ANOVA (Delay × Group) revealed a main effect of Delay [*F*(4, 112) = 144.5, *p* < 0.01] such that rats chose the large reward less frequently as the delay to reward delivery increased, as well as a main effect of Group [*F*(1, 28) = 6.59, *p* = 0.02] and a delay × group interaction [*F*(4, 112) = 3.52, *p* < 0.01], such that RE rats showed reduced preference for the large reward, particularly at short delays (Figure 4A). There was a notable gap between the RN and RE females’ choice of the large reward in the first block (0 s delay), which was driven by a cluster of RE females (*n* = 5 out of 14) avoiding the large reward even in the absence of delay to its delivery (less than 80% choice of the large reward at the 0 s delay). Removing these rats (as well as one similar outlier from the RN group) closed the gap at the 0 s delay but preserved the significant delay × group interaction [*F*(4, 88) = 2.5, *p* = 0.05], with RE females still choosing the large delayed reward less frequently than RN females, most notably at the 10 and 20 s delays (data not shown). This result might indicate greater sensitivity of RE females to the delay cost associated with the large reward, as the indifference point (50% choice of large delayed reward) for this subgroup of RE females (individuals with more than 80% choice of the large reward at the 0 s delay) was at 10.5 s versus 21.4 s for the RN group (Figure 4B).

Latencies to lever press were analyzed using a three-factor repeated-measures ANOVA (Group × Delay × Lever). On forced-choice trials, there were main effects of Delay [*F*(2.85, 159.8) = 21.2, *p* < 0.01] and Lever [*F*(1, 56) = 10.94, *p* < 0.01], as well as a Delay × Lever interaction [Delay × Lever: *F*(4, 224) = 29.43, *p* < 0.01], such that latencies were longer on both the large reward lever and at longer delays, and the difference in latencies between the two levers was larger at longer delays. There were no main effects or interactions involving Group, however [Group: *F*(1, 56) = 0.11, *p* = 0.74; Group × Delay: *F*(4, 224) = 0.81, *p* = 0.52; Group × Lever: *F*(1, 56) = 0.19, *p* = 0.67; Group × Lever × Delay: *F*(4, 224) = 1.19, *p* = 0.32]. Analyses of latencies on free-choice trials revealed results similar to those on forced-choice trials, with main effects of Delay [*F*(2.55, 139.6) = 55.01, *p* < 0.01] and Lever [*F*(1, 56) = 17.19, *p* < 0.01] and a Delay × Lever interaction [Delay × Lever: *F*(4, 219) = 15.75, *p* < 0.01], but no main effects or interactions involving Group [Group: *F*(1, 56) = 0.34, *p* = 0.56; Group × Delay: *F*(4, 219) = 0.78, *p* = 0.54; Group × Lever: *F*(1, 56) = 0.15, *p* = 0.7; Group × Lever × Delay: *F*(4, 219) = 0.39, *p* = 0.81].

A Welch’s *t*-test comparing locomotor activity in the intertemporal choice task showed no significant difference in activity between RE and RN females [*t*(25.85) = 0.16, *p* = 0.88]. The same analysis conducted on the number of omitted trials showed that RE females omitted fewer trials than RN females [*t*(21.55) = 2.50, *p* = 0.02]. Table 1 shows the mean (SEM) values for locomotor activity and omitted trials between female groups.

### 3.6 Reward omission vs. punishment task

To determine the effects of reproductive experience on decisions between two different types of risk, rats were tested in a novel behavioral task in which they chose between rewards of the same magnitude (one food pellet) that were associated with different costs (risk of reward omission vs. risk of punishment; the ROVP task).

**Figure 5:**
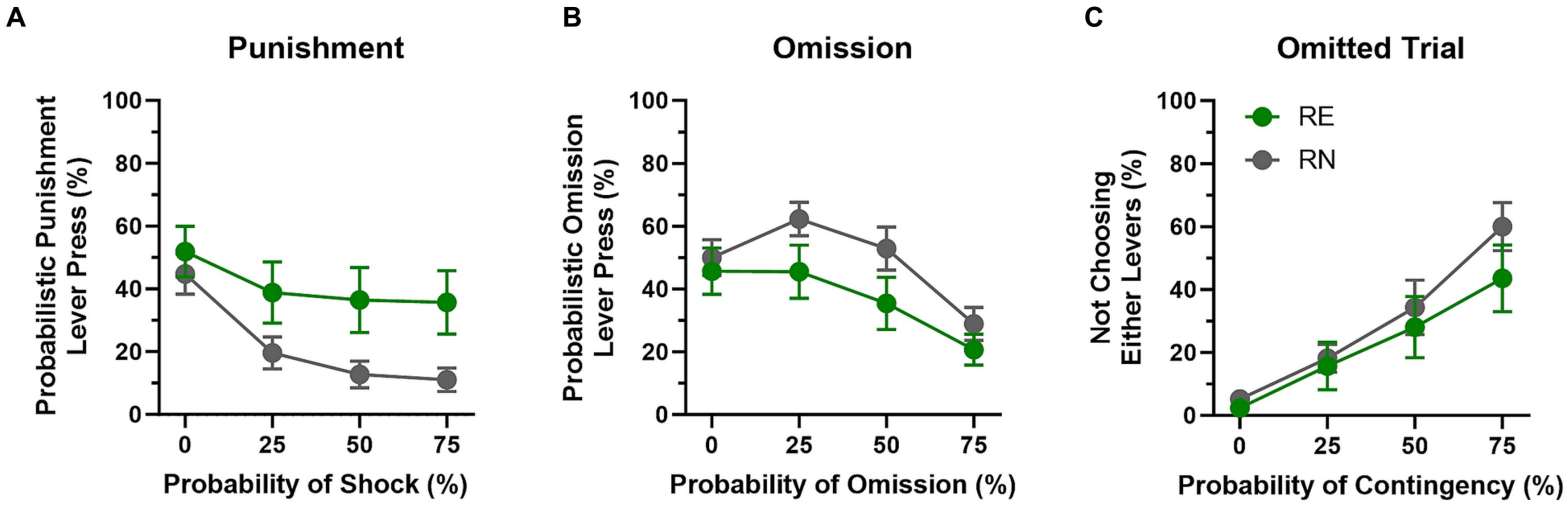
Reward omission vs. punishment task performance. **(A)** RE females chose the punishment option more frequently than RN females, but this difference was not statistically reliable. **(B)** There were no significant group differences in preference for the reward omission option. **(C)** The number of omitted trials increased across blocks, but was comparable between the two groups. Data are represented as means ± SEM.

Rats were tested on the ROVP task until stable behavior emerged (first cohort, 7 sessions; second cohort, 24 sessions; Figure 5). All rats chose the punished option less frequently as the probability of the contingencies increased across blocks of trials [*F*(1.54, 43.25) = 18.7, *p* < 0.01]. Choice of the punished option was numerically greater in RE compared to RN females, but this difference did not reach statistical significance [Group: *F*(1, 28) = 3.99, *p* = 0.06; Group × Probability: *F*(3, 84) = 2.27, *p* = 0.09]. Choice of the reward omission option also decreased across blocks of trials [*F*(1.88, 52.75) = 12.33, *p* < 0.01], but, as with choice of the punished option, it did not differ significantly between groups [Group: *F*(1, 28) = 2.79, *p* = 0.11; Group × Probability: *F*(3, 84) = 0.82, *p* = 0.49]. Finally, trial omissions increased substantially across blocks of trials [*F*(1.94, 54.28) = 36.50, *p* < 0.01], but also did not differ between groups [Group: *F*(1, 28) = 0.71, *p* = 0.41; Group × Probability: *F*(3, 84) = 0.92, *p* = 0.43].

Analyses of win-stay/lose-shift behavior in the ROVP task (two-factor repeated measures ANOVA, Group × Probability) revealed a main effect of reproductive experience on win-stay performance on the punishment side [Group, *F*(1, 20) = 4.95, *p* = 0.04], such that RE females repeated their choice of the punishment option after a win trial more frequently than RN females. No other significant main effects or interactions were observed with either the punishment or the reward omission option (Table 2).

**Table 2:** Win-stay/lose-shift performance in the ROVP.

	Punishment		Reward omission	
	Win-Stay	Lose-shift	Win-Stay	Lose-shift
Group	*F*(1, 20) = 4.95, *p* = 0.04	*F*(1, 20) = 1.73, *p* = 0.20	*F*(1, 28) = 0.21, *p* = 0.65	*F*(1, 28) = 1.18, *p* = 0.29
Probability of contingency	*F*(1.83, 21.06) = 0.94, *p* = 0.92	*F*(1.71, 25.63) = 1.55, *p* = 0.23	*F*(1.99, 42.8) = 1, *p* = 0.97	*F*(1.87, 46.66) = 1.12, *p* = 0.33
Group × probability of contingency	*F*(2, 23) = 0.45, *p* = 0.65	*F*(2, 30) = 0.11, *p* = 0.89	*F*(2, 43) = 0.74, *p* = 0.48	*F*(2, 50) = 0.88, *p* = 0.42

## 4 Discussion

Plasticity of the female brain during pregnancy and post-partum is widely recognized, yet literature on long term effects of reproductive experience on executive functions is scarce, particularly in animal models in which environmental variables can be tightly controlled. Here we show that the full spectrum of reproductive experience (mating, pregnancy, parturition, and pup rearing) is associated with performance differences across multiple cost–benefit decision making tasks in female rats. RE females showed greater preference for large, probabilistically punished over small, safe rewards, and reduced preference for large, delayed over small, immediate rewards.

### 4.1 Risky decision making

In the RDT, RE females showed greater preference than RN females for the large reward accompanied by probabilistic punishment (i.e., greater risk taking). This difference was not likely due to diminished sensitivity to footshock, as shock reactivity was equivalent in the two groups. There were further no differences between groups in the progressive ratio task, nor were there differences in probabilistic reversal learning, suggesting that differences in food motivation or cognitive flexibility also did not account for the greater risk taking in the RE group. These conclusions are supported by the results from the intertemporal choice task, in which RE females showed reduced preference for large rewards, as well as a more rapid shift toward preference for the small, immediate reward across trial blocks (see Intertemporal Choice section below for additional discussion). Interestingly, RE females’ pattern of choices in the ROVP task (greater preference for the probabilistically punished option compared to RN females) was analogous to that in the RDT, although this difference in the ROVP task did not reach statistical significance.

Risky decision making in general, and performance in the RDT specifically, are strongly regulated by dopamine signaling (Orsini et al., 2015; Piantadosi et al., 2021; França and Pompeia, 2023). Previous work with the RDT shows that higher levels of risk taking are associated with both higher levels of evoked dopamine release in nucleus accumbens (Freels et al., 2020) and lower expression of D2 receptor mRNA in striatum (Mitchell et al., 2014). Dopamine signaling is also implicated in some aspects of reproduction-related behavior, including food cravings during pregnancy, maternal behavior, and post-partum depression (Robinson et al., 2011; Rincón-Cortés and Grace, 2020, 2022; Haddad-Tóvolli et al., 2022). Recordings of dopamine availability from nucleus accumbens in rats using fast-scan cyclic voltammetry show dopamine transients in mothers when interacting with pups, and that evoked dopamine release under anesthesia is greater in early post-partum compared to reproductively-naïve rats (Robinson et al., 2011; Shnitko et al., 2017). These findings are consistent with data showing greater sensitivity to dopamine agonists in post-partum compared to reproductively-naïve rats (Byrnes et al., 2001, 2011). In humans, PET imaging data show reduced D2/3 receptor availability in striatum post-partum (Moses-Kolko et al., 2012). Given the similarities in dopamine signaling between rats with higher levels of risk taking behavior and post-partum subjects, it seems likely that reproduction-induced shifts in dopamine signaling play a causal role in the elevated risk taking observed in RE females.

In addition to changes in dopamine signaling, the post-partum period is associated with changes in circulating levels of ovarian hormones, including reductions in estradiol and prolactin (Bridges and Byrnes, 2006), some of which can persist for up to 3 years in humans (Barrett et al., 2014). Interestingly, ovariectomy (which reduces estradiol levels) causes an increase in risk taking in the RDT, which is partially reversed by estradiol replacement (Orsini et al., 2021). Although the mechanisms by which these manipulations of ovarian hormones cause alterations in risk taking are unclear, it is possible that they act at least in part through shifts in dopamine signaling linked to altered prolactin and estrogen levels (Bridges and Byrnes, 2006).

### 4.2 Intertemporal choice

In the intertemporal choice task, RE females showed reduced preference for the large reward relative to RN females, particularly at shorter (including zero) delays to delivery. Failures to reliably choose the large reward in the absence of delays in intertemporal choice tasks can be interpreted as a deficit in sensitivity to reward magnitude or reduced motivation to obtain the large reward. Inspection of individual rats’ performance at the 0 s delay, however, revealed that when rats from both RE and RN groups that chose the large reward on fewer than 80% of trials at the 0 s delay were excluded, there was equivalent choice of the large reward between the two groups in this block, but the significant interaction between group and delay remained. This (admittedly post-hoc) analysis suggests that reduced preference for the large, delayed reward in RE females is not solely due to reduced sensitivity to the large reward, but also reflects greater preference for small, immediate over large, delayed reward (i.e., greater impulsive choice). Consistent with this interpretation, RE females omitted fewer trials than RN females in this task and showed intact preference for the large reward in the RDT, not to mention the absence of a difference between the two groups in the progressive ratio task.

Performance on the intertemporal choice task and the RDT is not correlated in rats (Simon et al., 2009; Olshavsky et al., 2014; Shimp et al., 2015; Orsini and Setlow, 2017), but elevated levels of both risk taking and preference for immediate over delayed gratification are associated in some clinical conditions (e.g., substance use disorders; Bornovalova et al., 2005; Smith and Cyders, 2016; Costanza et al., 2021). The fact that RE females showed this same pattern of performance in the two tasks relative to RN females could suggest that a common mechanism underlies both changes. Postpartum shifts in dopamine signaling are one possible candidate for such changes. Lower levels of striatal D2/3 receptor availability are associated with greater discounting of delayed rewards in several clinical populations (Heinz et al., 2004; Ballard et al., 2015; Joutsa et al., 2015) and similar findings are evident in untreated rats (Dalley et al., 2007; Joutsa et al., 2015). Given that striatal D2/3 receptor availability is reduced in postpartum women (Moses-Kolko et al., 2012), such changes in dopamine signaling could account for the shifts in both risky and intertemporal choice observed in the present study.

### 4.3 Reproductive experience and aging

Animal models of aging, similar to other fields of animal research, use reproductively-naive subjects as standard practice, but the extent to which this represents the human condition (in which many individuals have experience with at least some aspect of reproductive experience) remains to be addressed. Reproductive history has been recognized as a contributing factor to cognitive aging outcomes in humans (Harville et al., 2020; Jung et al., 2020; Orchard et al., 2023). For example, number of childbirths is a predictor of brain age, with parous women exhibiting “younger-looking” brains in middle age (de Lange et al., 2019). Using MRI data, the same investigators found that more childbirths were associated with less apparent brain aging based on structural characteristic (cortical thickness, area, and volume) in striatal and limbic regions and, in particular, the nucleus accumbens (de Lange et al., 2020). Longer reproductive span and greater number of children are also associated with larger gray matter volume in brain regions vulnerable to Alzheimer’s disease and cognitive aging in midlife (Schelbaum et al., 2021). Such findings emphasize the importance of including reproductive experience in aging studies.

### 4.4 Limitations and future directions

One limitation of the current findings concerns the task designs, in which the contingencies in the RDT, intertemporal choice task, and ROVP task were set to an ascending order (i.e., increasing delays or probability of punishment). This issue is important as, in some cases, the same manipulation can have opposite effects on choice behavior in block design decision-making tasks such as those used here, depending on whether the contingencies increase or decrease across blocks (St Onge et al., 2010; Orsini et al., 2017b, 2018). Such differences in the results of manipulations depending on the order in which choice contingencies are presented have been interpreted as effects on the ability to adapt choices in the context of contingency changes (i.e., behavioral flexibility). As the direction of the effects of reproductive experience was not consistent across tasks (i.e., increases vs. decreases in choice of large, costly rewards in RE females), nor were there significant group differences on the probabilistic reversal learning task, it is unlikely that differences in behavioral flexibility fully account for the effects of reproductive experience on decision making. Nevertheless, future studies should address this issue more directly. Another potential limitation concerns possible changes in pain sensitivity in RE females. Although RE and RN females did not differ in shock reactivity, pain is a multi-dimensional construct that is expressed via multiple types of behavior mediated by distinct levels of the neuraxis (Melzack and Casey, 1968; Garland, 2012; Case et al., 2016). Although previous work showed that risk taking in the RDT is unrelated to several measures of pain sensitivity, this work was conducted only in male rats (Simon et al., 2011). It will thus be important in future work to conduct more thorough assessments of pain/shock sensitivity, particularly because parturition is known to cause increases in pain tolerance that can persist well beyond postpartum (Berlit et al., 2018), and low striatal D2/3 receptor availability predicts high pain threshold in healthy individuals (Pertovaara et al., 2004; Martikainen et al., 2018).

Female rats can become pregnant while nursing, and thus it is likely that estrous cycles had resumed by the time behavioral testing commenced (3 weeks after the second weaning; Bridges and Byrnes, 2006). As previous work has shown no relationships in reproductively-naïve rats between estrous phase and cost–benefit decision making (Orsini et al., 2016; Hernandez et al., 2020), it is unlikely that estrous cycle had large effects on performance in the current experiments. This said, because reproductive experience can exert some long-lasting effects on hypothalamic–pituitary-gonadal signaling (Bridges and Byrnes, 2006; Barrett et al., 2014), it is possible that relationships between estrous phase and task performance (as well as its underlying dopaminergic mechanisms) were altered in RE females. As such, it will be useful to incorporate estrous cycle measurements in future work comparing RE and RN females. It should also be noted that the present work does not distinguish which specific aspect(s) of females’ reproductive experience might be responsible for the observed behavioral differences between groups. Future experiments are required to elucidate the contributions of each of the reproductive experience components to altered decision making in RE females.

One way in which to view the more impulsive and risky choices observed in RE females could be from the perspective of care for offspring. The increased preference for immediate gratification and greater willingness to take risks to obtain a larger food reward could be interpreted as a (long-lasting) shift in behavioral strategy that might help to facilitate feeding and protecting offspring (perhaps analogous to the emergence of maternal aggression). Finally, given that performance in both the intertemporal choice task and the RDT is associated with elements of executive function such as working memory and set shifting (Shimp et al., 2015; Hernandez et al., 2017), it would be useful to determine in future work the extent to which effects of reproductive experience on behavior extend to other aspects of PFC-mediated cognition.

## 5 Summary and conclusion

The results of these studies demonstrate a suite of alterations in decision making in RE females (greater risk taking and impulsive choice) that could shed light on post-partum changes in psychiatric disorders, given established links between such psychiatric disorders and both risky and intertemporal choice (Klein et al., 2008; Swann et al., 2008; Takahashi et al., 2008; Pailing and Reniers, 2018; Deborah et al., 2020; Johnson et al., 2022). Additional studies are needed to determine the physiological and neurobiological mechanisms of these effects (e.g., via changes in gonadal hormone and/or dopamine signaling), particularly because the vast majority of such work has focused on short post-partum timescales. More importantly, however, given the prevalence of reproductive experience in humans, these data suggest that such experience should be considered as a variable in preclinical models of human conditions such as aging, in which (at least in women), it has been linked to differential risks for brain volumetric changes and even Alzheimer’s disease (Fox et al., 2018; Bae et al., 2020; Jung et al., 2020; Schelbaum et al., 2021).

## Data availability statement

The raw data supporting the conclusions of this article will be made available by the authors, without undue reservation.

## Ethics statement

The animal study was approved by the University of Florida Institutional Animal Care and Use Committee. The study was conducted in accordance with the local legislation and institutional requirements.

## Author contributions

MF: Writing – review & editing, Conceptualization, Data curation, Formal analysis, Investigation, Writing – original draft. OV-R: Writing – review & editing, Data curation. BS: Writing – review & editing, Supervision, Conceptualization, Funding acquisition, Project administration. JB: Supervision, Writing – review & editing, Funding acquisition, Project administration.

## References

[ref1] Albin-Brooks C. Nealer C. Sabihi S. Haim A. Leuner B. (2017). The influence of offspring, parity, and oxytocin on cognitive flexibility during the postpartum period. Horm. Behav. 89, 130–136. doi: 10.1016/j.yhbeh.2016.12.015, PMID: 28062230 PMC5986067

[ref2] Alcántara-Alonso V. Panetta P. de Gortari P. Grammatopoulos D. K. (2017). Corticotropin-releasing hormone as the homeostatic rheostat of Feto-maternal symbiosis and developmental programming in utero and neonatal life. Front. Endocrinol. 8:161. doi: 10.3389/fendo.2017.00161, PMID: 28744256 PMC5504167

[ref3] Amlung M. Marsden E. Holshausen K. Morris V. Patel H. Vedelago L.. (2019). Delay discounting as a transdiagnostic process in psychiatric disorders: a meta-analysis. JAMA Psychiatry 76, 1176–1186. doi: 10.1001/jamapsychiatry.2019.2102, PMID: 31461131 PMC6714026

[ref4] Arévalo L. Campbell P. (2020). Placental effects on the maternal brain revealed by disrupted placental gene expression in mouse hybrids. Proc. Biol. Sci. 287:20192563. doi: 10.1098/rspb.2019.2563 31937228 PMC7003458

[ref5] Bae J. B. Lipnicki D. M. Han J. W. Sachdev P. S. Kim T. H. Kwak K. P.. (2020). Does parity matter in women's risk of dementia? A COSMIC collaboration cohort study. BMC Med. 18:210. doi: 10.1186/s12916-020-01671-1, PMID: 32753059 PMC7406389

[ref6] Bak Y. Nah Y. Han S. Lee S.-K. Shin N.-Y. (2020). Altered neural substrates within cognitive networks of postpartum women during working memory process and resting-state. Sci. Rep. 10:9110. doi: 10.1038/s41598-020-66058-x, PMID: 32499565 PMC7272423

[ref7] Ballard M. E. Mandelkern M. A. Monterosso J. R. Hsu E. Robertson C. L. Ishibashi K.. (2015). Low dopamine D2/D3 receptor availability is associated with steep discounting of delayed rewards in methamphetamine dependence. Int. J. Neuropsychopharmacol. 18:pyu119. doi: 10.1093/ijnp/pyu119, PMID: 25603861 PMC4540098

[ref8] Barba-Müller E. Craddock S. Carmona S. Hoekzema E. (2019). Brain plasticity in pregnancy and the postpartum period: links to maternal caregiving and mental health. Arch. Womens Ment. Health 22, 289–299. doi: 10.1007/s00737-018-0889-z, PMID: 30008085 PMC6440938

[ref9] Barrett E. S. Parlett L. E. Windham G. C. Swan S. H. (2014). Differences in ovarian hormones in relation to parity and time since last birth. Fertil. Steril. 101, 1773–80.e1. doi: 10.1016/j.fertnstert.2014.02.047, PMID: 24684956 PMC4041832

[ref10] Berlit S. Lis S. Häfner K. Kleindienst N. Baumgärtner U. Treede R.-D.. (2018). Changes in birth-related pain perception impact of neurobiological and psycho-social factors. Arch. Gynecol. Obstet. 297, 591–599. doi: 10.1007/s00404-017-4605-4, PMID: 29196870

[ref11] Bernal A. Paolieri D. (2022). The influence of estradiol and progesterone on neurocognition during three phases of the menstrual cycle: modulating factors. Behav. Brain Res. 417:113593. doi: 10.1016/j.bbr.2021.113593, PMID: 34560130

[ref12] Bhatia P. Chhabra S. (2018). Physiological and anatomical changes of pregnancy: implications for anaesthesia. Indian J. Anaesth. 62, 651–657. doi: 10.4103/ija.IJA_458_18, PMID: 30237589 PMC6144551

[ref13] Blaes S. L. Shimp K. G. Betzhold S. M. Setlow B. Orsini C. A. (2022). Chronic cocaine causes age-dependent increases in risky choice in both males and females. Behav. Neurosci. 136, 243–263. doi: 10.1037/bne0000509, PMID: 35298207 PMC9346435

[ref14] Bonnet K. A. Peterson K. E. (1975). A modification of the jump-flinch technique for measuring pain sensitivity in rats. Pharmacol. Biochem. Behav. 3, 47–55. doi: 10.1016/0091-3057(75)90079-9, PMID: 1129354

[ref15] Bornovalova M. A. Daughters S. B. Hernandez G. D. Richards J. B. Lejuez C. W. (2005). Differences in impulsivity and risk-taking propensity between primary users of crack cocaine and primary users of heroin in a residential substance-use program. Exp. Clin. Psychopharmacol. 13, 311–318. doi: 10.1037/1064-1297.13.4.311, PMID: 16366761

[ref16] Bridges R. S. Byrnes E. M. (2006). Reproductive experience reduces circulating 17β-estradiol and prolactin levels during Proestrus and alters estrogen sensitivity in female rats. Endocrinology 147, 2575–2582. doi: 10.1210/en.2005-0917, PMID: 16484327

[ref17] Brunton P. J. (2019). Endogenous opioid signalling in the brain during pregnancy and lactation. Cell Tissue Res. 375, 69–83. doi: 10.1007/s00441-018-2948-1, PMID: 30415283

[ref18] Brunton P. J. Russell J. A. (2008). The expectant brain: adapting for motherhood. Nat. Rev. Neurosci. 9, 11–25. doi: 10.1038/nrn2280 18073776

[ref19] Byrnes J. J. Bridges R. S. Byrnes E. M. (2011). Amphetamine sensitization in reproductively experienced female rats. Neurosci. Lett. 502, 168–172. doi: 10.1016/j.neulet.2011.07.035, PMID: 21821097 PMC3167002

[ref20] Byrnes E. M. Byrnes J. J. Bridges R. S. (2001). Increased sensitivity of dopamine systems following reproductive experience in rats. Pharmacol. Biochem. Behav. 68, 481–489. doi: 10.1016/S0091-3057(01)00449-X, PMID: 11325402

[ref21] Cáceda R. Nemeroff C. B. Harvey P. D. (2014). Toward an understanding of decision making in severe mental illness. J. Neuropsychiatry Clin. Neurosci. 26, 196–213. doi: 10.1176/appi.neuropsych.12110268, PMID: 24599051

[ref22] Cárdenas E. F. Kujawa A. Humphreys K. L. (2020). Neurobiological changes during the peripartum period: implications for health and behavior. Soc. Cogn. Affect. Neurosci. 15, 1097–1110. doi: 10.1093/scan/nsz091, PMID: 31820795 PMC7657461

[ref23] Case L. K. Laubacher C. M. Olausson H. Wang B. Spagnolo P. A. Bushnell M. C. (2016). Encoding of touch intensity but not pleasantness in human primary somatosensory cortex. J. Neurosci. 36, 5850–5860. doi: 10.1523/JNEUROSCI.1130-15.2016, PMID: 27225773 PMC4879201

[ref24] Chechko N. Dukart J. Tchaikovski S. Enzensberger C. Neuner I. Stickel S. (2022). The expectant brain–pregnancy leads to changes in brain morphology in the early postpartum period. Cereb. Cortex 32, 4025–4038. doi: 10.1093/cercor/bhab463, PMID: 34942007 PMC9476604

[ref25] Costanza A. Rothen S. Achab S. Thorens G. Baertschi M. Weber K.. (2021). Impulsivity and impulsivity-related Endophenotypes in suicidal patients with substance use disorders: an exploratory study. Int. J. Ment. Heal. Addict. 19, 1729–1744. doi: 10.1007/s11469-020-00259-3

[ref26] Dalley J. W. Fryer T. D. Brichard L. Robinson E. S. Theobald D. E. Lääne K.. (2007). Nucleus accumbens D2/3 receptors predict trait impulsivity and cocaine reinforcement. Science 315, 1267–1270. doi: 10.1126/science.1137073, PMID: 17332411 PMC1892797

[ref27] Dalton G. L. Phillips A. G. Floresco S. B. (2014). Preferential involvement by nucleus accumbens shell in mediating probabilistic learning and reversal shifts. J. Neurosci. 34, 4618–4626. doi: 10.1523/JNEUROSCI.5058-13.2014, PMID: 24672007 PMC6608131

[ref28] de Lange A. G. Barth C. Kaufmann T. Anatürk M. Suri S. Ebmeier K. P.. (2020). The maternal brain: region-specific patterns of brain aging are traceable decades after childbirth. Hum. Brain Mapp. 41, 4718–4729. doi: 10.1002/hbm.25152, PMID: 32767637 PMC7555081

[ref29] de Lange A.-M. G. Kaufmann T. van der Meer D. Maglanoc L. Alnæs D. Moberget T.. (2019). Population-based neuroimaging reveals traces of childbirth in the maternal brain. BioRxiv 116, 22341–22346. doi: 10.1073/pnas.1910666116 PMC6825266 31615888

[ref30] Deborah A. C.-C. Sarah C. D. Nathan K. (2020). Depression, risk preferences and risk-taking behavior. J. Hum. Resour. 57, 1566–1160. doi: 10.3368/jhr.58.1.0419-10183R1

[ref31] Duarte-Guterman P. Leuner B. Galea L. A. M. (2019). The long and short term effects of motherhood on the brain. Front. Neuroendocrinol. 53:100740. doi: 10.1016/j.yfrne.2019.02.004, PMID: 30826374

[ref32] Eid R. S. Chaiton J. A. Lieblich S. E. Bodnar T. S. Weinberg J. Galea L. A. M. (2019). Early and late effects of maternal experience on hippocampal neurogenesis, microglia, and the circulating cytokine milieu. Neurobiol. Aging 78, 1–17. doi: 10.1016/j.neurobiolaging.2019.01.021, PMID: 30825663

[ref33] Evenden J. L. Ryan C. N. (1996). The pharmacology of impulsive behaviour in rats: the effects of drugs on response choice with varying delays of reinforcement. Psychopharmacology 128, 161–170. doi: 10.1007/s002130050121, PMID: 8956377

[ref34] Fox M. Berzuini C. Knapp L. A. Glynn L. M. (2018). Women's pregnancy life history and Alzheimer's risk: can Immunoregulation explain the link? Am. J. Alzheimers Dis. Other Dement. 33, 516–526. doi: 10.1177/1533317518786447, PMID: 30060670 PMC6442681

[ref35] França T. F. A. Pompeia S. (2023). Reappraising the role of dopamine in adolescent risk-taking behavior. Neurosci. Biobehav. Rev. 147:105085. doi: 10.1016/j.neubiorev.2023.105085, PMID: 36773751

[ref36] Freels T. G. Gabriel D. B. K. Lester D. B. Simon N. W. (2020). Risky decision-making predicts dopamine release dynamics in nucleus accumbens shell. Neuropsychopharmacology 45, 266–275. doi: 10.1038/s41386-019-0527-0, PMID: 31546248 PMC6901435

[ref37] Garland E. L. (2012). Pain processing in the human nervous system: a selective review of nociceptive and biobehavioral pathways. Prim. Care 39, 561–571. doi: 10.1016/j.pop.2012.06.013, PMID: 22958566 PMC3438523

[ref38] Gowin J. L. Mackey S. Paulus M. P. (2013). Altered risk-related processing in substance users: imbalance of pain and gain. Drug Alcohol Depend. 132, 13–21. doi: 10.1016/j.drugalcdep.2013.03.019, PMID: 23623507 PMC3748224

[ref39] Haddad-Tóvolli R. Ramírez S. Muñoz-Moreno E. Milà-Guasch M. Miquel-Rio L. Pozo M.. (2022). Food craving-like episodes during pregnancy are mediated by accumbal dopaminergic circuits. Nat. Metab. 4, 424–434. doi: 10.1038/s42255-022-00557-1, PMID: 35379970

[ref40] Hahn-Holbrook J. Holt-Lunstad J. Holbrook C. Coyne S. M. Lawson E. T. (2011). Maternal defense: breast feeding increases aggression by reducing stress. Psychol. Sci. 22, 1288–1295. doi: 10.1177/0956797611420729, PMID: 21873570 PMC3345316

[ref41] Harris M. MacMillan H. Andrews K. Atkinson L. Kimber M. England-Mason G.. (2021). Maternal adverse childhood experiences, executive function and emotional availability in mother-child dyads. Child Abuse Negl. 111:104830. doi: 10.1016/j.chiabu.2020.104830, PMID: 33307519

[ref42] Harville E. W. Guralnik J. Romero M. Bazzano L. A. (2020). Reproductive history and cognitive aging: the Bogalusa heart study. Am. J. Geriatr. Psychiatry 28, 217–225. doi: 10.1016/j.jagp.2019.07.002, PMID: 31350162 PMC6942641

[ref43] Heinz A. Siessmeier T. Wrase J. Hermann D. Klein S. Grüsser S. M.. (2004). Correlation between dopamine D(2) receptors in the ventral striatum and central processing of alcohol cues and craving. Am. J. Psychiatry 161, 1783–1789. doi: 10.1176/ajp.161.10.1783 15465974

[ref44] Hernandez C. M. Orsini C. Wheeler A.-R. Ten Eyck T. W. Betzhold S. M. Labiste C. C.. (2020). Testicular hormones mediate robust sex differences in impulsive choice in rats. Elife 9:e58604. doi: 10.7554/eLife.58604, PMID: 32985975 PMC7521924

[ref45] Hernandez C. M. Vetere L. M. Orsini C. A. McQuail J. A. Maurer A. P. Burke S. N.. (2017). Decline of prefrontal cortical-mediated executive functions but attenuated delay discounting in aged Fischer 344 × brown Norway hybrid rats. Neurobiol. Aging 60, 141–152. doi: 10.1016/j.neurobiolaging.2017.08.025, PMID: 28946018 PMC5669385

[ref46] Hoekzema E. Barba-Müller E. Pozzobon C. Picado M. Lucco F. García-García D.. (2017). Pregnancy leads to long-lasting changes in human brain structure. Nat. Neurosci. 20, 287–296. doi: 10.1038/nn.4458, PMID: 27991897

[ref47] Hoekzema E. van Steenbergen H. Straathof M. Beekmans A. Freund I. M. Pouwels P. J. W.. (2022). Mapping the effects of pregnancy on resting state brain activity, white matter microstructure, neural metabolite concentrations and grey matter architecture. Nat. Commun. 13:6931. doi: 10.1038/s41467-022-33884-8, PMID: 36414622 PMC9681770

[ref48] Hoyt L. T. Falconi A. M. (2015). Puberty and perimenopause: reproductive transitions and their implications for women's health. Soc. Sci. Med. 132, 103–112. doi: 10.1016/j.socscimed.2015.03.031, PMID: 25797100 PMC4400253

[ref49] Johnson A. C. Cipolla M. J. (2015). The cerebral circulation during pregnancy: adapting to preserve normalcy. Physiology 30, 139–147. doi: 10.1152/physiol.00048.2014, PMID: 25729059 PMC5504451

[ref50] Johnson S. L. Porter P. A. Modavi K. Dev A. S. Pearlstein J. G. Timpano K. R. (2022). Emotion-related impulsivity predicts increased anxiety and depression during the COVID-19 pandemic. J. Affect. Disord. 301, 289–299. doi: 10.1016/j.jad.2022.01.037, PMID: 35026359 PMC8747782

[ref51] Joutsa J. Voon V. Johansson J. Niemelä S. Bergman J. Kaasinen V. (2015). Dopaminergic function and intertemporal choice. Transl. Psychiatry 5:e491. doi: 10.1038/tp.2014.133, PMID: 25562841 PMC4312827

[ref52] Jung J. H. Lee G. W. Lee J. H. Byun M. S. Yi D. Jeon S. Y.. (2020). Multiparity, brain atrophy, and cognitive decline. Front. Aging Neurosci. 12:159. doi: 10.3389/fnagi.2020.00159, PMID: 32581769 PMC7291884

[ref53] Kaye W. H. Wierenga C. E. Bailer U. F. Simmons A. N. Bischoff-Grethe A. (2013). Nothing tastes as good as skinny feels: the neurobiology of anorexia nervosa. Trends Neurosci. 36, 110–120. doi: 10.1016/j.tins.2013.01.003, PMID: 23333342 PMC3880159

[ref54] Kim P. Dufford A. J. Tribble R. C. (2018). Cortical thickness variation of the maternal brain in the first 6 months postpartum: associations with parental self-efficacy. Brain Struct. Funct. 223, 3267–3277. doi: 10.1007/s00429-018-1688-z, PMID: 29855765 PMC6358213

[ref55] Kinsley C. H. Amory-Meyer E. (2011). Why the maternal brain? J. Neuroendocrinol. 23, 974–983. doi: 10.1111/j.1365-2826.2011.02194.x 21790810

[ref56] Klein H. Elifson K. W. Sterk C. E. (2008). Depression and HIV risk behavior practices among at risk women. Women Health 48, 167–188. doi: 10.1080/03630240802313605, PMID: 19042215 PMC6192253

[ref57] Leuner B. Fredericks P. J. Nealer C. Albin-Brooks C. (2014). Chronic gestational stress leads to depressive-like behavior and compromises medial prefrontal cortex structure and function during the postpartum period. PLoS One 9:e89912. doi: 10.1371/journal.pone.0089912, PMID: 24594708 PMC3940672

[ref58] Leuner B. Glasper E. R. Gould E. (2010). Parenting and plasticity. Trends Neurosci. 33, 465–473. doi: 10.1016/j.tins.2010.07.003, PMID: 20832872 PMC3076301

[ref59] Linnet J. Møller A. Peterson E. Gjedde A. Doudet D. (2011). Inverse association between dopaminergic neurotransmission and Iowa gambling task performance in pathological gamblers and healthy controls. Scand. J. Psychol. 52, 28–34. doi: 10.1111/j.1467-9450.2010.00837.x, PMID: 20704689

[ref60] Maeng L. Y. Shors T. J. (2012). Once a mother, always a mother: maternal experience protects females from the negative effects of stress on learning. Behav. Neurosci. 126, 137–141. doi: 10.1037/a0026707, PMID: 22181714 PMC3279153

[ref61] Martikainen I. K. Hagelberg N. Jääskeläinen S. K. Hietala J. Pertovaara A. (2018). Dopaminergic and serotonergic mechanisms in the modulation of pain: in vivo studies in human brain. Eur. J. Pharmacol. 834, 337–345. doi: 10.1016/j.ejphar.2018.07.038, PMID: 30036531

[ref62] Martínez-García M. Paternina-Die M. Barba-Müller E. Martín de Blas D. Beumala L. Cortizo R.. (2021). Do pregnancy-induced brain changes reverse? The brain of a mother six years after parturition. Brain Sci. 11:168. doi: 10.3390/brainsci11020168, PMID: 33525512 PMC7912216

[ref63] Mastorakos G. Ilias I. (2000). Maternal hypothalamic-pituitary-adrenal axis in pregnancy and the postpartum period. Postpartum-related disorders. Ann. N. Y. Acad. Sci. 900, 95–106. doi: 10.1111/j.1749-6632.2000.tb06220.x, PMID: 10818396

[ref64] Meltzer-Brody S. Howard L. M. Bergink V. Vigod S. Jones I. Munk-Olsen T.. (2018). Postpartum psychiatric disorders. Nat. Rev. Dis. Primers. 4:18022. doi: 10.1038/nrdp.2018.22 29695824

[ref65] Melzack R. Casey K. (1968). “Sensory, motivational, and central control determinants of pain” in The skin senses. ed. Kenshalo D. R. (Springfield, Illinois: Charles C Thomas), 423–439.

[ref66] Miller E. S. Hoxha D. Wisner K. L. Gossett D. R. (2015a). Obsessions and compulsions in postpartum women without obsessive compulsive disorder. J Womens Health 24, 825–830. doi: 10.1089/jwh.2014.5063 26121364

[ref67] Miller E. S. Hoxha D. Wisner K. L. Gossett D. R. (2015b). The impact of perinatal depression on the evolution of anxiety and obsessive-compulsive symptoms. Arch. Womens Ment. Health 18, 457–461. doi: 10.1007/s00737-014-0476-x, PMID: 25355541 PMC7082147

[ref68] Mitchell S. H. (2019). Linking delay discounting and substance use disorders: genotypes and phenotypes. Perspect. Behav. Sci. 42, 419–432. doi: 10.1007/s40614-019-00218-x 31976442 PMC6768927

[ref69] Mitchell M. R. Weiss V. G. Beas B. S. Morgan D. Bizon J. L. Setlow B. (2014). Adolescent risk taking, cocaine self-administration, and striatal dopamine signaling. Neuropsychopharmacology 39, 955–962. doi: 10.1038/npp.2013.295, PMID: 24145852 PMC3924529

[ref70] Mizuno T. Tamakoshi K. Tanabe K. (2017). Anxiety during pregnancy and autonomic nervous system activity: a longitudinal observational and cross-sectional study. J. Psychosom. Res. 99, 105–111. doi: 10.1016/j.jpsychores.2017.06.006, PMID: 28712414

[ref71] Morgan J. K. Santosa H. Fridley R. M. Conner K. K. Hipwell A. E. Forbes E. E.. (2021). Postpartum depression is associated with altered neural connectivity between affective and Mentalizing regions during mother-infant interactions. Front. Glob. Womens Health 2:744649. doi: 10.3389/fgwh.2021.744649 34816247 PMC8593996

[ref72] Morris R. W. (2018). “Chapter 17- goal-directed deficits in schizophrenia” in Goal-directed decision making academic press. eds. Morris R. Bornstein A. Shenhav A., 387–406.

[ref73] Moses-Kolko E. L. Banihashemi L. Hipwell A. E. (2021). Reduced postpartum hippocampal volume is associated with positive mother-infant caregiving behavior. J. Affect. Disord. 281, 297–302. doi: 10.1016/j.jad.2020.12.014, PMID: 33341012 PMC8950103

[ref74] Moses-Kolko E. L. Price J. C. Wisner K. L. Hanusa B. H. Meltzer C. C. Berga S. L.. (2012). Postpartum and depression status are associated with lower [11C] raclopride BPND in reproductive-age women. Neuropsychopharmacology 37, 1422–1432. doi: 10.1038/npp.2011.328, PMID: 22257897 PMC3327847

[ref75] Mukherjee D. Lee S. Kazinka R. Satterthwaite T. D. Kable J. W. (2020). Multiple facets of value-based decision making in major depressive disorder. Sci. Rep. 10:3415. doi: 10.1038/s41598-020-60230-z, PMID: 32099062 PMC7042239

[ref76] Nordenswan E. Deater-Deckard K. Karrasch M. Laine M. Kataja E. L. Holmberg E.. (2021). Maternal executive functioning, emotional availability and psychological distress during toddlerhood: a Finn brain birth cohort study. Front. Psychol. 12:735734. doi: 10.3389/fpsyg.2021.735734, PMID: 34690890 PMC8533223

[ref77] Olshavsky M. E. Shumake J. Rosenthal A. A. Kaddour-Djebbar A. Gonzalez-Lima F. Setlow B.. (2014). Impulsivity, risk-taking, and distractibility in rats exhibiting robust conditioned orienting behaviors. J. Exp. Anal. Behav. 102, 162–178. doi: 10.1002/jeab.104, PMID: 25130520

[ref78] Orchard E. R. Rutherford H. J. V. Holmes A. J. Jamadar S. D. (2023). Matrescence: lifetime impact of motherhood on cognition and the brain. Trends Cogn. Sci. 27, 302–316. doi: 10.1016/j.tics.2022.12.002, PMID: 36609018 PMC9957969

[ref79] Orsini C. A. Blaes S. L. Hernandez C. M. Betzhold S. M. Perera H. Wheeler A. R.. (2021). Regulation of risky decision making by gonadal hormones in males and females. Neuropsychopharmacology 46, 603–613. doi: 10.1038/s41386-020-00827-0, PMID: 32919406 PMC8027379

[ref80] Orsini C. A. Hernandez C. M. Singhal S. Kelly K. B. Frazier C. J. Bizon J. L.. (2017a). Optogenetic inhibition reveals distinct roles for basolateral amygdala activity at discrete time points during risky decision making. J. Neurosci. 37, 11537–11548. doi: 10.1523/JNEUROSCI.2344-17.2017, PMID: 29079687 PMC5707761

[ref81] Orsini C. A. Heshmati S. C. Garman T. S. Wall S. C. Bizon J. L. Setlow B. (2018). Contributions of medial prefrontal cortex to decision making involving risk of punishment. Neuropharmacology 139, 205–216. doi: 10.1016/j.neuropharm.2018.07.018, PMID: 30009836 PMC6108435

[ref82] Orsini C. A. Mitchell M. R. Heshmati S. C. Shimp K. G. Spurrell M. S. Bizon J. L.. (2017b). Effects of nucleus accumbens amphetamine administration on performance in a delay discounting task. Behav. Brain Res. 321, 130–136. doi: 10.1016/j.bbr.2017.01.001, PMID: 28057530 PMC5272779

[ref83] Orsini C. A. Moorman D. E. Young J. W. Setlow B. Floresco S. B. (2015). Neural mechanisms regulating different forms of risk-related decision-making: insights from animal models. Neurosci. Biobehav. Rev. 58, 147–167. doi: 10.1016/j.neubiorev.2015.04.009, PMID: 26072028 PMC7913606

[ref84] Orsini C. A. Setlow B. (2017). Sex differences in animal models of decision making. J. Neurosci. Res. 95, 260–269. doi: 10.1002/jnr.23810, PMID: 27870448 PMC5120608

[ref85] Orsini C. A. Willis M. L. Gilbert R. J. Bizon J. L. Setlow B. (2016). Sex differences in a rat model of risky decision making. Behav. Neurosci. 130, 50–61. doi: 10.1037/bne0000111, PMID: 26653713 PMC4738105

[ref86] Osborne L. M. Payne J. L. Sherer M. L. Sabunciyan S. (2022). Altered extracellular mRNA communication in postpartum depression is associated with decreased autophagy. Mol. Psychiatry 27, 4526–4535. doi: 10.1038/s41380-022-01794-2, PMID: 36138128

[ref87] Pailing A. N. Reniers R. (2018). Depressive and socially anxious symptoms, psychosocial maturity, and risk perception: associations with risk-taking behaviour. PLoS One 13:e0202423. doi: 10.1371/journal.pone.0202423, PMID: 30110384 PMC6093696

[ref88] Pawluski J. L. Lonstein J. S. Fleming A. S. (2017). The neurobiology of postpartum anxiety and depression. Trends Neurosci. 40, 106–120. doi: 10.1016/j.tins.2016.11.009 28129895

[ref89] Pawluski J. L. Vanderbyl B. L. Ragan K. Galea L. A. M. (2006). First reproductive experience persistently affects spatial reference and working memory in the mother and these effects are not due to pregnancy or ‘mothering’ alone. Behav. Brain Res. 175, 157–165. doi: 10.1016/j.bbr.2006.08.017 17011053

[ref90] Pertovaara A. Martikainen I. K. Hagelberg N. Mansikka H. Någren K. Hietala J.. (2004). Striatal dopamine D2/D3 receptor availability correlates with individual response characteristics to pain. Eur. J. Neurosci. 20, 1587–1592. doi: 10.1111/j.1460-9568.2004.03622.x, PMID: 15355325

[ref91] Piantadosi P. T. Halladay L. R. Radke A. K. Holmes A. (2021). Advances in understanding meso-cortico-limbic-striatal systems mediating risky reward seeking. J. Neurochem. 157, 1547–1571. doi: 10.1111/jnc.15342, PMID: 33704784 PMC8981567

[ref92] Purcell J. R. Herms E. N. Morales J. Hetrick W. P. Wisner K. M. Brown J. W. (2022). A review of risky decision-making in psychosis-spectrum disorders. Clin. Psychol. Rev. 91:102112. doi: 10.1016/j.cpr.2021.102112 34990988 PMC8754677

[ref93] Putnick D. L. Sundaram R. Bell E. M. Ghassabian A. Goldstein R. B. Robinson S. L.. (2020). Trajectories of maternal postpartum depressive symptoms. Pediatrics 146:e20200857. doi: 10.1542/peds.2020-0857 33109744 PMC7772818

[ref94] Rehbein E. Kogler L. Kotikalapudi R. Sattler A. Krylova M. Kagan K. O.. (2022). Pregnancy and brain architecture: associations with hormones, cognition and affect. J. Neuroendocrinol. 34:e13066. doi: 10.1111/jne.13066, PMID: 35014110

[ref95] Reyes L. M. Usselman C. W. Davenport M. H. Steinback C. D. (2018). Sympathetic nervous system regulation in human normotensive and hypertensive pregnancies. Hypertension 71, 793–803. doi: 10.1161/HYPERTENSIONAHA.117.10766, PMID: 29531177

[ref96] Rincón-Cortés M. Grace A. A. (2020). Adaptations in reward-related behaviors and mesolimbic dopamine function during motherhood and the postpartum period. Front. Neuroendocrinol. 57:100839. doi: 10.1016/j.yfrne.2020.100839, PMID: 32305528 PMC7531575

[ref97] Rincón-Cortés M. Grace A. A. (2022). Dopamine downregulation in novel rodent models useful for the study of postpartum depression. Front. Behav. Neurosci. 16:1065558. doi: 10.3389/fnbeh.2022.1065558, PMID: 36620861 PMC9812956

[ref98] Robinson D. L. Zitzman D. L. Williams S. K. (2011). Mesolimbic dopamine transients in motivated behaviors: focus on maternal behavior. Front. Psych. 2:23. doi: 10.3389/fpsyt.2011.00023 PMC3098725 21629844

[ref99] Roes M. Galea L. A. M. (2016). “Chapter 9- the maternal brain: short-and long-term effects of reproductive experience on Hippocampus structure and function in adulthood” in Sex differences in the central nervous system. ed. Shansky R. M. (San Diego: Academic Press), 197–220.

[ref100] Schelbaum E. Loughlin L. Jett S. Zhang C. Jang G. Malviya N.. (2021). Association of reproductive history with brain MRI biomarkers of dementia risk in midlife. Neurology 97:e2328. doi: 10.1212/WNL.0000000000012941 34732544 PMC8665431

[ref101] Servin-Barthet C. Martínez-García M. Pretus C. Paternina-Pie M. Soler A. Khymenets O.. (2023). The transition to motherhood: linking hormones, brain and behaviour. Nat. Rev. Neurosci. 24, 605–619. doi: 10.1038/s41583-023-00733-6, PMID: 37612425

[ref102] Shimp K. G. Mitchell M. R. Beas B. S. Bizon J. L. Setlow B. (2015). Affective and cognitive mechanisms of risky decision making. Neurobiol. Learn. Mem. 117, 60–70. doi: 10.1016/j.nlm.2014.03.002, PMID: 24642448 PMC4164592

[ref103] Shnitko T. A. Mace K. D. Sullivan K. M. Martin W. K. Andersen E. H. Williams Avram S. K.. (2017). Use of fast-scan cyclic voltammetry to assess phasic dopamine release in rat models of early postpartum maternal behavior and neglect. Behav. Pharmacol. 28, 648–660. doi: 10.1097/FBP.0000000000000347 29068793 PMC5680131

[ref104] Shors T. J. (2016). A trip down memory lane about sex differences in the brain. Philos. Trans. R. Soc. B Biol. Sci. 371:20150124. doi: 10.1098/rstb.2015.0124, PMID: 26833842 PMC4785907

[ref105] Simon N. W. Gilbert R. J. Mayse J. D. Bizon J. L. Setlow B. (2009). Balancing risk and reward: a rat model of risky decision making. Neuropsychopharmacology 34, 2208–2217. doi: 10.1038/npp.2009.48, PMID: 19440192 PMC2726909

[ref106] Simon N. W. Montgomery K. S. Beas B. S. Mitchell M. R. LaSarge C. L. Mendez I. A.. (2011). Dopaminergic modulation of risky decision-making. J. Neurosci. 31, 17460–17470. doi: 10.1523/JNEUROSCI.3772-11.2011, PMID: 22131407 PMC3307370

[ref107] Skalkidou A. Hellgren C. Comasco E. Sylvén S. Sundström Poromaa I. (2012). Biological aspects of postpartum depression. Womens Health 8, 659–672. doi: 10.2217/WHE.12.55 23181531

[ref108] Smith G. T. Cyders M. A. (2016). Integrating affect and impulsivity: the role of positive and negative urgency in substance use risk. Drug Alcohol Depend. 163, S3–S12. doi: 10.1016/j.drugalcdep.2015.08.038, PMID: 27306729 PMC4911536

[ref109] Soutschek A. Jetter A. Tobler P. N. (2022). Toward a unifying account of Dopamine’s role in cost-benefit decision making. Biol. Psychiatry Glob. Open Sci. 3, 179–186. doi: 10.1016/j.bpsgos.2022.02.010 37124350 PMC10140448

[ref110] St Onge J. R. Chiu Y. C. Floresco S. B. (2010). Differential effects of dopaminergic manipulations on risky choice. Psychopharmacology 211, 209–221. doi: 10.1007/s00213-010-1883-y 20495787

[ref111] Steinglass J. E. Lempert K. M. Choo T.-H. Kimeldorf M. B. Wall M. Walsh B. T.. (2017). Temporal discounting across three psychiatric disorders: anorexia nervosa, obsessive compulsive disorder, and social anxiety disorder. Depress. Anxiety 34, 463–470. doi: 10.1002/da.22586, PMID: 28009473 PMC5869031

[ref112] Swann A. C. Steinberg J. L. Lijffijt M. Moeller F. G. (2008). Impulsivity: differential relationship to depression and mania in bipolar disorder. J. Affect. Disord. 106, 241–248. doi: 10.1016/j.jad.2007.07.011, PMID: 17822778 PMC2683682

[ref113] Takahashi T. Oono H. Inoue T. Boku S. Kako Y. Kitaichi Y.. (2008). Depressive patients are more impulsive and inconsistent in intertemporal choice behavior for monetary gain and loss than healthy subjects--an analysis based on Tsallis' statistics. Neuro Endocrinol. Lett. 29, 351–358. PMID: 18580849

[ref114] Tal R. Taylor H. S. (2000). “Endocrinology of pregnancy” in Endotext. eds. Feingold K. R. Anawalt B. Boyce A. Chrousos G. Herder W. W. Dhatariya K.. (South Dartmouth, MA: MDText.com, Inc)

[ref115] Tan E. K. Tan E. L. (2013). Alterations in physiology and anatomy during pregnancy. Best Pract. Res. Clin. Obstet. Gynaecol. 27, 791–802. doi: 10.1016/j.bpobgyn.2013.08.001 24012425

[ref116] Teng C. Otero M. Geraci M. Blair R. J. Pine D. S. Grillon C.. (2016). Abnormal decision-making in generalized anxiety disorder: aversion of risk or stimulus-reinforcement impairment? Psychiatry Res. 237, 351–356. doi: 10.1016/j.psychres.2015.12.031, PMID: 26822065 PMC4988522

[ref117] Valyan A. Ekhtiari H. Smith R. Paulus M. P. (2020). “Chapter 4- decision-making deficits in substance use disorders: cognitive functions, assessment paradigms, and levels of evidence” in Cognition and addiction. ed. Verdejo-Garcia A. (Cambridge, Massachusetts: Academic Press), 25–61.

[ref118] Walter M. H. Abele H. Plappert C. F. (2021). The role of oxytocin and the effect of stress during childbirth: neurobiological basics and implications for mother and child. Front. Endocrinol. 12:742236. doi: 10.3389/fendo.2021.742236 PMC8578887 34777247

[ref119] Wen L. Li R. Wang J. Yi J. (2019). The reproductive stress hypothesis. Reproduction 158, R209–r218. doi: 10.1530/REP-18-0592, PMID: 31677601 PMC6892456

[ref120] Worthen R. J. Beurel E. (2022). Inflammatory and neurodegenerative pathophysiology implicated in postpartum depression. Neurobiol. Dis. 165:105646. doi: 10.1016/j.nbd.2022.105646, PMID: 35104645 PMC8956291

